# Repetitive Head Movements: An Unusual Subcortical Myoclonus Presentation

**DOI:** 10.1055/a-2846-8189

**Published:** 2026-05-06

**Authors:** Morgana Fregonese, Davide Caputo, Marco Moscatelli, Federica Rachele Danti, Giovanna Zorzi, Federica Graziola

**Affiliations:** 1 Department of Biomedical and Clinical Sciences, Postgraduate School of Child Neuropsychiatry, University of Milan, Milan, Italy; 2 Department of Paediatric Neuroscience, Fondazione IRCCS Istituto Neurologico Carlo Besta, Milan, Italy; 3 Neuroradiology Unit, Fondazione IRCCS Istituto Neurologico Carlo Besta, Milan, Italy

We present a 4-year-old boy referred to our hospital for repetitive head movements following a history of right cerebellar ganglioglioma (grade I WHO 2016, BRAF V600E positive), partially surgically removed at 24 months of age. Eleven months after surgery, he began to exhibit almost-continuous movements of right flexion and left rotation of the head, present while awake but absent during sleep. No signs of cranial nerve weakness were observed at clinical examination. Several treatment attempts, including Clonazepam, Valproic Acid, Tetrabenazine, and Levodopa (L-Dopa), yielded no clinical improvement. Botulinum toxin injection was considered but not performed at the choice of the family.

**Fig. 1:**
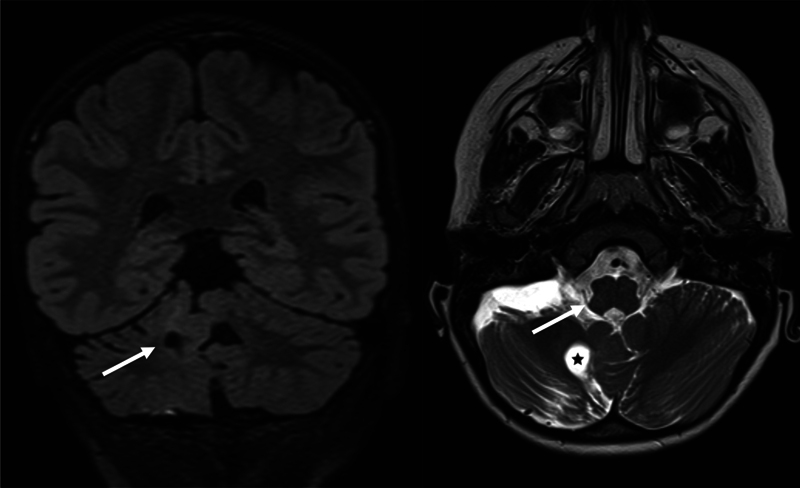
Brain MRI. (Left) Coronal FLAIR shows the extension of pathological tissue to the right dentate nucleus (arrow), apex of Mollaret's triangle. (Right) Axial T2. The bulb shows normal morphology and signal. The surgical cavity (star) is distant from the course of the accessory nerve (arrow).

Brain MRI revealed the stability of the area with mild contrast enhancement in the right cerebellar and ponto-mesencephalic region, attributed to residual disease. No abnormalities were detected in the medulla oblongata, and iatrogenic lesions of the accessory nerve were excluded ( Fig. 1 ).

Electrophysiological studies identified the presence of very low-frequency (0.5–0.2 Hz) irregular EMG bursts involving the right sternocleidomastoid and trapezium muscles, lasting 150 milliseconds each ( Video 1 ). The clinical and neurophysiological characteristics of this “oscillatory” pattern suggest a diagnosis of subcortical myoclonus consistent with bulbar/upper spinal generator or spinal nerve involvement. 1 This case mimics a rare movement disorder, Holmes tremor, characterized by a combination of rest, posture, and action high amplitude and low frequency tremor, typically affecting the limbs, arising from various underlying structural disorders (usually trauma or stroke) affecting the triangle of Guillain and Mollaret. 2 In our patients, the underlying pathophysiology remains similar, 3 with the extension of pathological tissue to the right dentate nucleus, apex of Mollaret's triangle. The correct localization of the generator of the phenomenon remains intriguing, as in our case, the residual lesion involves the cerebellar and ponto-mesencephalic regions, while the medulla oblongata and the XI nerve seem spared from the lesion and surgery. However, the timing of the appearance of the phenomenon is consistent with an iatrogenic origin. 4

**Video 1** Video EEG polygraphic recording very low-frequency (0.5–0.2 Hz), irregular EMG bursts involving the right sternocleidomastoid and trapezium muscles, lasting 150 milliseconds each. These hyperkinetic movements were present irrespective of patient activity, both at rest and during postural activation or head movements, and were unresponsive to distraction or submaximal forced contraction of the neck. No EEG correlate was observed at the back-averaging analysis. EMG1 right splenius capitis muscle, EMG2 left splenius capitis muscle, EMG3 right sternocleidomastoid, EMG4 left sternocleidomastoid.


This case highlights the importance of integrating clinical, neurophysiological, and imaging data to refine movement disorder classification.
